# Nomogram predictive model for the incidence and risk factors of persistent fever after cardiovascular surgery

**DOI:** 10.1186/s12893-025-03161-8

**Published:** 2025-10-09

**Authors:** Feng Zang, Guangxu Mao, Ziyao Quan, Yongfeng Shao, Sheng Zhao, Liyun Wang, Zhanjie Li, Zhongqiu You, Lu Liu, Wensen Chen

**Affiliations:** 1 Department of Infection Management, The First Affiliated Hospital of Nanjing Medical University, Nanjing, 210029 Jiangsu China; 2 Department of Infection Management, Xinghua People’s Hospital Affiliated to Yangzhou University, Xinghua, 225700 Jiangsu China; 3 Department of Disease Control and Prevention, General Hospital of Eastern Theatre Command, Nanjing, 210002 Jiangsu China; 4 Department of Cardiovascular Surgery, The First Affiliated Hospital with Nanjing Medical University, Nanjing, 210029 Jiangsu China; 5 Department of Infection Management, Chengdu Pidu District People’s Hospital, Chengdu, 610000 Sichuan China; 6 Department of Infection Management, Yizheng People’s Hospital, Yangzhou, 211400 Jiangsu China

**Keywords:** Cardiovascular surgery, Persistent postoperative fever, Risk factors, Nomogram predictive model

## Abstract

**Background:**

A persistent fever following cardiovascular surgery presents a significant clinical challenge and often leads to adverse patient outcomes. This study aims to develop a nomogram predictive model for persistent postoperative fever, which could serve as a valuable tool for clinicians in making diagnostic and treatment decisions.

**Methods:**

The medical records of patients who underwent cardiovascular surgery at the First Affiliated Hospital of Nanjing Medical University in 2023 were retrospectively analysed. The patients were divided into two groups based on whether their body temperature remained above 38℃ for three consecutive days after surgery: the persistent fever group and the control group. Independent risk factors for persistent postoperative fever were identified through univariate and multivariate logistic regression analyses. A predictive nomogram model was then developed and validated.

**Results:**

The study involved 343 patients who underwent cardiovascular surgery, revealing an overall postoperative fever rate of 70.55% and a persistent fever rate of 38.78%. The highest fever rates were observed on the first and second postoperative days. Multivariate logistic regression analysis identified several independent risk factors for persistent postoperative fever, including older age at admission, a history of smoking, a higher Controlling Nutritional Status Score (CONUT), an elevated Monocyte-to-Lymphocyte Ratio (MLR), a longer duration of surgery, the use of cardiopulmonary bypass, and intraoperative transfusion of machine-washed red blood cells. A nomogram prediction model showed good discriminatory and predictive ability, with an area under the receiver operating characteristic curve (AUC) of 0.821 (95% CI: 0.775–0.866).

**Conclusion:**

The nomogram developed in this research offers a quantitative and visually intuitive method for early evaluation of the probability of persistent postoperative fever in individuals undergoing cardiovascular surgery.

Cardiovascular disease continues to be a leading cause of global morbidity and mortality, with about 17.9 million deaths in 2019, representing 32% of all global deaths according to the World Health Organisation (WHO) [1]. Major cardiovascular surgeries are frequently necessary to save lives in these cases. Despite progress in surgical techniques and post-operative care, patients undergoing such procedures are still at risk for various complications, with fever being one of the most common [2]. Fever is a frequent occurrence following cardiovascular surgery, impacting around 60–70% of patients [3]. The intricate nature of cardiac surgery, which involves substantial tissue damage, triggers a series of pro-inflammatory reactions that may result in a temporary state of immune suppression [4]. At the same time, non-infectious causes such as the surgical stress response, thromboembolism, wound infections, drug or transfusion reactions, and the inflammatory consequences of cardiopulmonary bypass (CPB) are common causes of postoperative fever [5]. Although early postoperative fever is typically short-lived and not indicative of infection, a fever that persists or develops later may signal infection, inflammation, or other complications, and is associated with poorer clinical outcomes. The Medical Information Mart for Intensive Care (MIMIC)-IV database indicates that a fever persisting after abdominal surgery, particularly within the first few days post-operation, correlates with a significantly extended stay in the intensive care unit (ICU) and increased ICU mortality [6]. A cohort study involving 6,122 cardiac surgery patients found that while high fever within 24 h postoperatively did not significantly impact short- or long-term mortality, persistent high fever markedly prolonged both ICU and hospital stays [7]. In patients experiencing a fever lasting more than 48 h after cardiac surgery, the risk of infectious complications, including wound infections, catheter-related infections, pneumonia, and other conditions, increased significantly. This highlights the importance of early intervention. These interventions may involve symptom-specific treatments, antibiotic courses (if bacterial infections are confirmed or suspected), and diagnostic procedures like blood and urine cultures. The study aimed to analyse the risk factors associated with persistent fever following cardiovascular surgery. Identifying these factors is crucial for enhancing patient outcomes, reducing hospital stays, and optimising the use of medical resources.

## Subjects and methods

### Study subjects

**Fig. 1:**
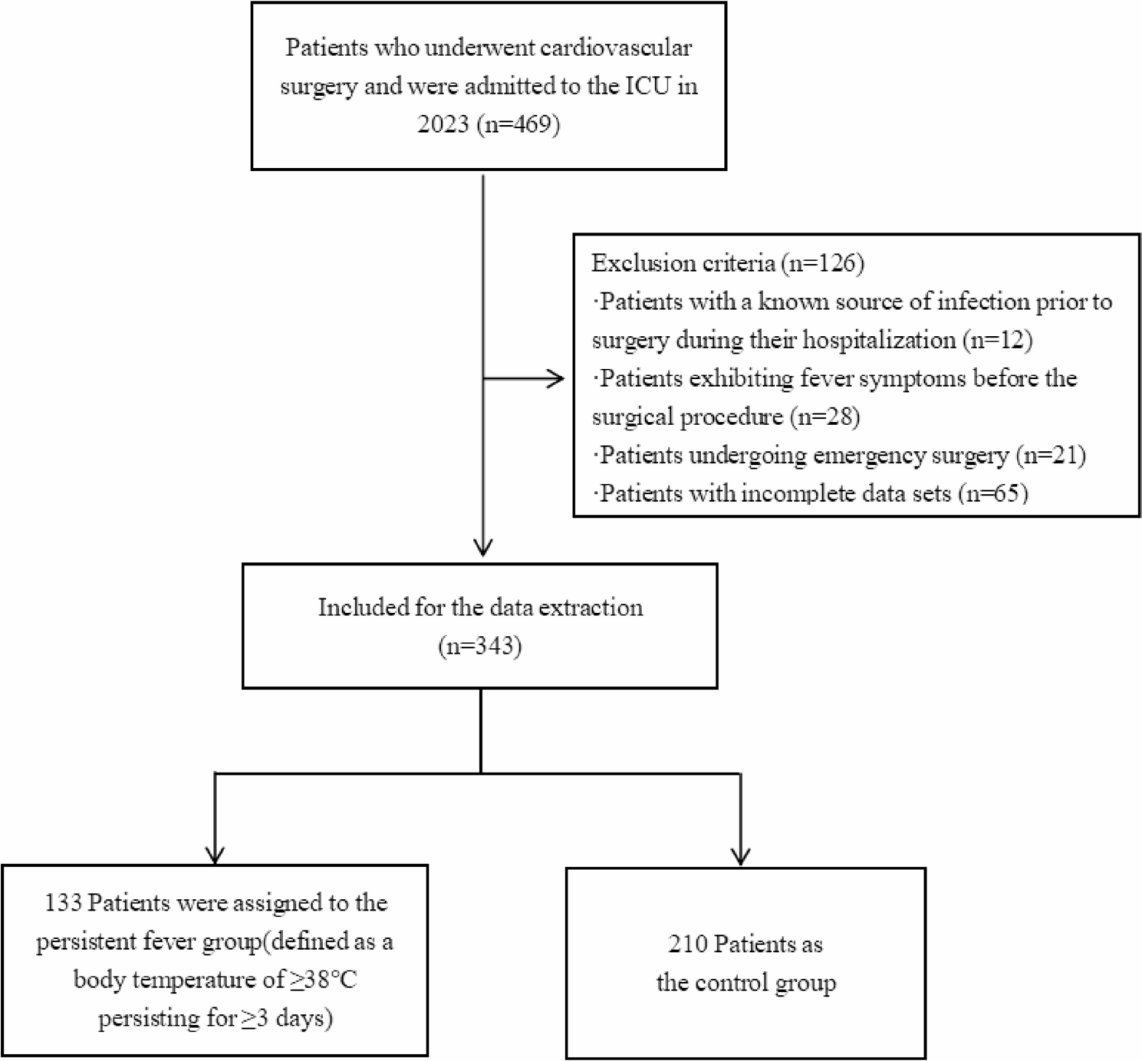
Flow chart of participants selection

The study focused on patients who had cardiovascular surgery and were admitted to the ICU at the First Affiliated Hospital of Nanjing Medical University in 2023. A random sample of 469 patients was initially chosen, but after applying rigorous exclusion criteria, only 343 patients met the final enrollment criteria. Exclusion criteria were clearly defined as follows: (1) patients with a known source of infection before surgery during their hospitalisation; (2) patients exhibiting fever symptoms before the surgical procedure; (3) patients undergoing emergency surgery; and (4) patients with incomplete data sets. Subsequently, the enrolled patients were categorised into two groups: those with persistent fever (defined as a body temperature of ≥ 38℃ persisting for ≥ 3 days) and a control group without this fever criterion. Figure 1 shows the flow chart for participant selection.

### Research methods

#### General information

Clinical data including age, gender, height, weight, smoking history, alcohol consumption history, underlying diseases (hypertension, diabetes), intraoperative details (type of surgery, cardiopulmonary bypass records, duration of surgery, intraoperative blood loss and blood product transfusion, ASA score, NNIS score, intraoperative medication, etc.) were gathered. Smoking and alcohol consumption history in this study pertains to a consistent pattern of smoking and alcohol intake within one month before hospital admission. Laboratory test data included preoperative serum albumin (ALB), total cholesterol (TC), monocytes (MONO), absolute neutrophil count (ANC), lymphocyte count (ALC), platelet count (PLT), and haemoglobin (Hb). oral temperature measurement is typically employed preoperatively, while postoperative temperature is dynamically monitored using a temperature-measuring urinary catheter to assess bladder temperature. The postoperative body temperature of 343 patients was meticulously recorded in the electronic body temperature record form within the medical records. We define the daily fever rate as the percentage of patients with a body temperature of 38℃ or higher on postoperative day n, calculated by dividing the number of such patients by the total number of patients and multiplying by 100%. The persistent fever rate is defined as the percentage of patients exhibiting a body temperature of 38℃ or higher for three consecutive days postoperatively, similarly calculated. The study was approved by the Ethics Committee of the First Affiliated Hospital of Nanjing Medical University(2024-SR-5

#### Nutritional and inflammatory markers [8, 9]

The nutritional and inflammatory status of patients was assessed using their initial blood test results upon admission. Nutritional biomarkers included the Prognostic Nutritional Index (PNI) and the Controlling Nutritional Status (CONUT). Inflammatory biomarkers encompassed the monocyte-to-lymphocyte ratio (MLR), the neutrophil-to-lymphocyte ratio (NLR), the platelet-to-lymphocyte ratio (PLR), and the systemic inflammatory index (SII). The calculation formulas and evaluation criteria are detailed in Table 1.

**Table 1:** Inflammatory and nutritional markers utilised in the study

Laboratory indicator	Calculation method			
Inflammatory markers:				
Monocyte to lymphocyte ratio (MLR)	total number of monocytes/total number of lymphocytes			
Neutrophil to lymphocyte ratio (NLR)	total number of neutrophils/total number of lymphocytes			
Platelet to lymphocyte ratio (PLR)	total number of platelets/total number of lymphocytes			
Systemic immune-inflammation index (SII)	total number of neutrophils×total number of platelets/ total number of lymphocytes			
Nutritional markers:				
Prognostic Nutritional Index (PNI)	serum albumin (g/L) + 0.005×total number of lymphocytes(µL)			
Controlling nutritional status (CONUT):				
	Normal	Mild	Moderate	Severe
Albumin (g/L)	>=35	30-34.9	25-29.9	< 25
Score(ALB)	0	2	4	6
Total lymphocytes count(µL)	>=1600	1200–1590	800–1199	< 800
Score(TLC)	0	1	2	3
Total cholesterol (mmol/L)	>=4.68	3.64–4.67	2.60–3.63	< 2.60
Score(TC)	0	1	2	3
Total score	Score(ALB) + Score(TLC) + Score(TC)			

## Statistical methods

Statistical analysis of the data was conducted using SPSS 26.0 and R software (version 4.0.2). Measurement data following a normal distribution with homogeneous variance were presented as mean ± standard deviation, and independent sample t-tests were employed for comparing the two groups. Enumeration data analysis was presented as n (%), and χ2 tests were used to compare the two groups. Logistic regression models were utilised to investigate the potential risk factors associated with persistent fever following cardiovascular surgery. Receiver Operating Characteristic (ROC) curves were employed to assess the diagnostic accuracy of each variable. All statistical analyses were conducted using two-sided tests, with a significance level set at *P* < 0.05.

## Results

### Fever incidence

**Fig. 2:**
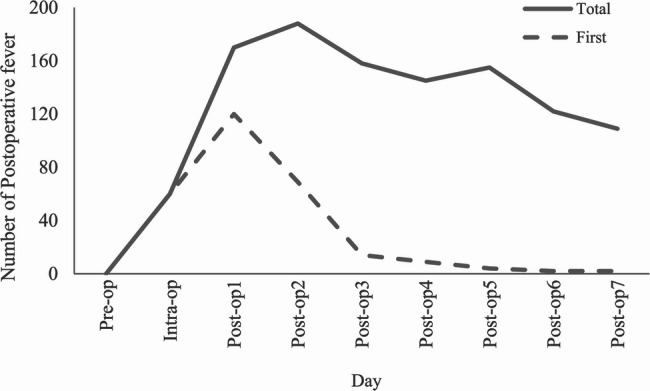
Cumulative and new cases of fever within 7 days after surgery

An analysis was conducted on the incidence of fever within seven days post-surgery among 343 patients. The results indicated that the overall incidence rate of fever during this period was 70.55% (242/343). The highest rates of fever were observed on the first and second days post-surgery, at 42.86% and 45.48%, respectively. Furthermore, 38.78% of patients experienced persistent fever following surgery. Figure 2 illustrates the number of patients with new or cumulative fever within seven days of surgery, among a total of 343 patients.

### Analysis of risk factors for persistent fever after cardiovascular surgery

**Fig. 3:**
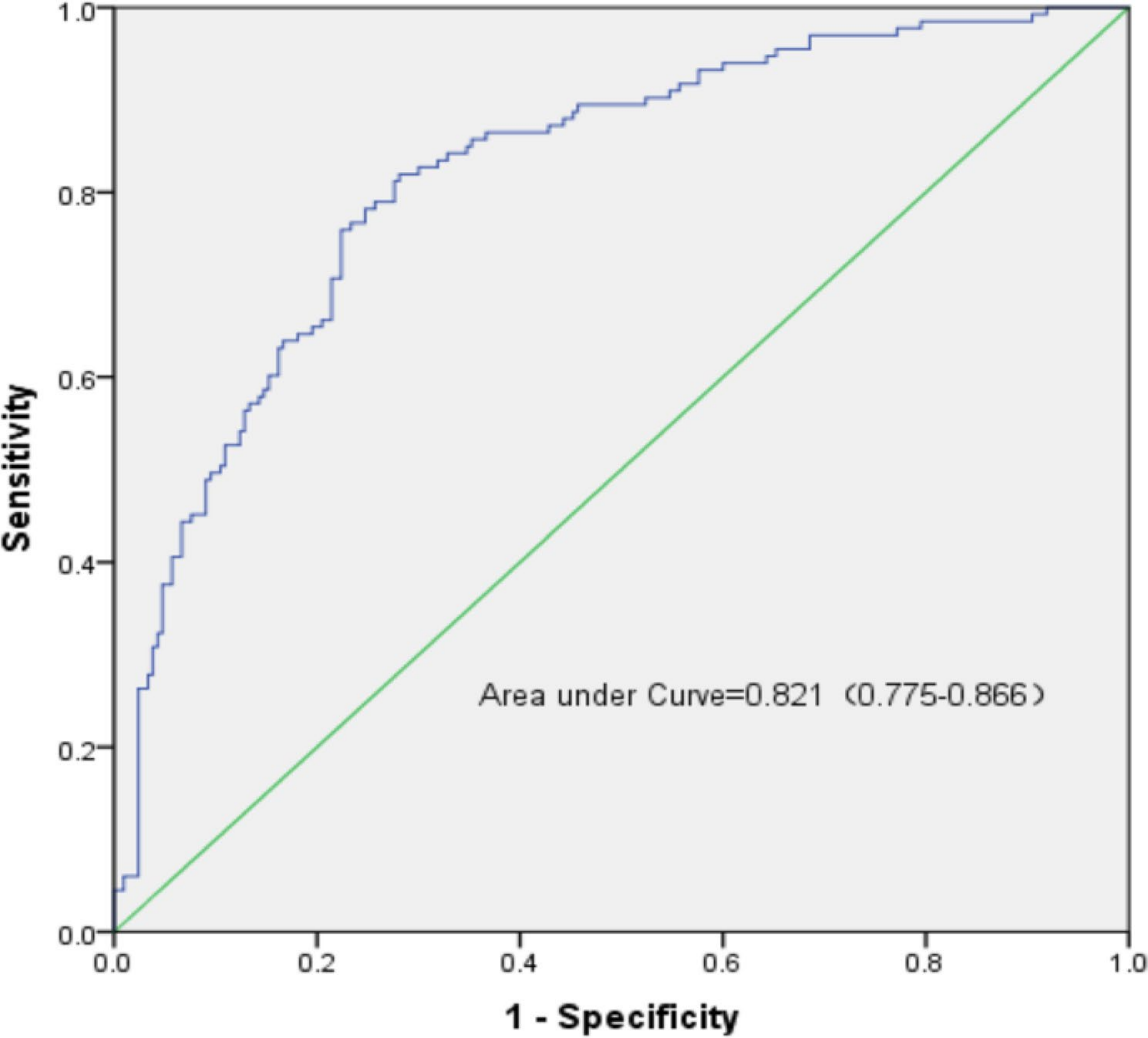
ROC curve of the nomogram prediction model

Univariate analysis revealed several risk factors for postoperative persistent fever, including age at admission, smoking history, drinking history, CONUT score, NLR score, MLR score, duration of surgery, cardiopulmonary bypass (CPB), ASA score, intraoperative transfusion of red blood cells, washed red blood cells, and platelets (*P* < 0.05) (refer to Table 2). Multivariate logistic regression analysis was conducted to identify independent predictors of postoperative persistent fever, revealing that age at admission, smoking history, CONUT score, MLR score, duration of surgery, CPB, and intraoperative autologous red blood cell transfusion were significant factors (refer to Table 3). The Hosmer-Lemeshow goodness-of-fit test indicated a good fit for the model (χ2 = 6.184, *P* > 0.05), suggesting strong predictive performance. Furthermore, the area under the ROC curve (AUC) was calculated as 0.821 (95% CI: 0.775–0.866), demonstrating the model’s good discriminative ability (see Fig. 3).

**Table 2:** Univariate analysis of persistent fever after cardiovascular surgery

Variable		Control	Persistent fever	x^2^/t	*P*
		(*n* = 210)	(*n* = 133)		
Baseline					
age		61.02 ± 10.66	63.74 ± 9.72	−2.375	0.018
sex	Female	96(45.71)	51(38.35)	1.805	0.179
	Male	114(54.29)	82(61.65)		
height(cm)		163.51 ± 7.69	165.43 ± 8.31	−1.82	0.07
smoking history	No	174(82.86)	85(63.91)	15.808	< 0.001
	Yes	36(17.14)	48(36.09)		
drinking history	No	178(84.76)	99(74.44)	5.587	0.018
	Yes	32(15.24)	34(25.56)		
diabetes	No	180(85.71)	105(78.95)	2.654	0.103
	Yes	30(14.29)	28(21.05)		
hypertension	No	133(63.33)	71(53.38)	3.345	0.067
	Yes	77(36.67)	62(46.62)		
Preoperative index					
CONUT		1.61 ± 1.36	2.56 ± 1.98	−4.864	< 0.001
PNI		49.56 ± 19.24	46.28 ± 5.45	1.912	0.057
NLR		2.23 ± 1.13	3.01 ± 3.01	−2.811	0.006
MLR		0.29 ± 0.13	0.37 ± 0.24	−3.492	0.001
PLR		120.63 ± 45.75	127.65 ± 61.75	−1.129	0.26
SII		425.04 ± 249.14	521.61 ± 562.08	−1.869	0.063
hemoglobin		131.79 ± 19.21	129.80 ± 20.76	0.902	0.368
Intraoperative information					
Operation duration		4.07 ± 1.61	5.34 ± 1.79	−6.78	< 0.001
cardiopulmonary bypass	No	70(33.33)	16(12.03)	19.671	< 0.001
	Yes	140(66.67)	117(87.97)		
operation	coronary artery	68(32.38)	34(25.56)	2.921	0.404
	cardiac valves	87(41.43)	62(46.62)		
	comprehensive operation	18(8.57)	16(12.03)		
	other	37(17.62)	21(15.79)		
ASA score	Ⅰ~Ⅱ	26(12.38)	6(4.51)	5.962	0.015
	Ⅲ~Ⅴ	184(87.62)	127(95.49)		
NNIS score	1	25(13.44)	10(8.93)	1.373	0.241
	2	161(86.56)	102(91.07)		
prophylactic	No	50(23.81)	22(16.54)	2.594	0.107
	Yes	160(76.19)	111(83.46)		
bleeding volume		383.38 ± 330.64	329.40 ± 367.36	1.411	0.159
Intraoperative autologous red blood cell transfusion	No	154(73.33)	84(63.16)	3.969	0.046
	Yes	56(26.67)	49(36.84)		
Leukocyte transfusion	No	112(53.33)	46(34.58)	11.518	0.001
	Yes	98(46.67)	87(65.41)		
Platelet transfusion	No	130(61.90)	56(42.11)	12.861	< 0.001

**Table 3:** Multivariate logistic regression for predictive equation

Variable		β	Standard errors	Wald	*P*	OR	95%CI
Age		0.031	0.014	5.235	0.022	1.032	1.004–1.060
Smoking history	No	1.104	0.301	13.474	< 0.001	3.015	1.672–5.434
	Yes						
CONURT score		0.266	0.093	8.133	0.004	1.305	1.087–1.567
MLR score		1.959	0.885	4.902	0.027	7.090	1.252–40.152
Operation duration		0.349	0.09	15.103	< 0.001	1.417	1.189–1.690
Cardiopulmonary bypass	No	1.305	0.366	12.702	< 0.001	3.687	1.799–7.555
	Yes						
Intraoperative autologous red blood cell transfusion	No	0.726	0.272	7.126	0.008	2.068	1.213–3.525
	Yes						
Cons		−6.943	1.078	41.478	< 0.001		

### Nomogram for risk of postoperative persistent fever

**Fig. 4:**
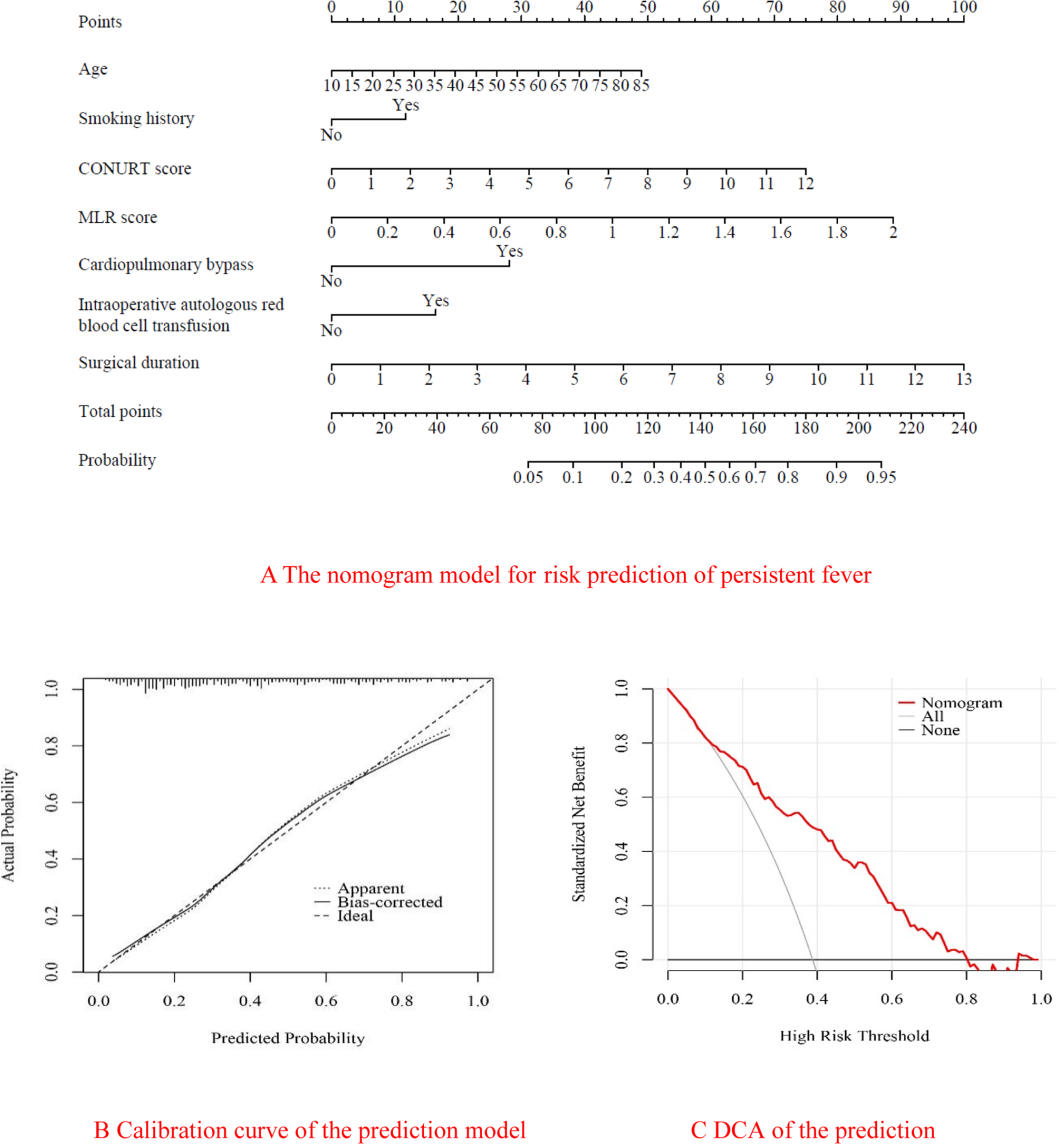
**A** The nomogram model for risk prediction of persistent fever. **B **Calibration curve of the prediction model. **C** DCA of the prediction

A nomogram was created to predict the risk of postoperative persistent fever based on the results of binary logistic regression analysis. In Fig. 4A, it is evident that the duration of surgery had the highest impact on the nomogram, followed by MLR, CONUT score, age, and CPB. Figure 4B illustrates the good calibration of the nomogram prediction model, with a mean absolute error of 0.016, a mean squared error of 0.00047, and a 0.9 quantile of absolute error of 0.029. The calibration curve of the model closely aligned with the reference line, demonstrating strong agreement between predicted and actual probabilities. Decision Curve Analysis (DCA) revealed threshold probabilities ranging from 0.11 to 0.8 (refer to Fig. 4C).

## Discussion

The study revealed that 70.55% of patients undergoing cardiovascular surgery experienced fever, with a persistent fever rate of 38.78%. These findings align with existing literature on fever rates post major surgeries, specifically cardiac procedures [3, 10]. A prospective study of 75 cardiac surgery patients revealed a postoperative fever incidence of approximately 64%. The study also noted that postoperative infections were infrequent, accounting for only 13%, and were typically caused by easily treatable pathogens [11]. Although fever is a common occurrence following cardiac surgery, the exact aetiology and mechanism are unknown. The presence of fever frequently prompts extensive diagnostic investigations, which may incur significant costs and pose potential risks to patients. Therefore, it is crucial to approach the evaluation of fever in a systematic, clinically sound, and cost-effective manner to prevent unnecessary interventions [2]. Related studies have shown that a considerable proportion of early postoperative fevers are non-infectious; therefore, the use of empirical antibiotics is not warranted unless there is a confirmed source of infection [12]. Previous research has primarily concentrated on identifying risk factors for postoperative fever following different types of surgeries, including non-infectious fever and postoperative infections [13]. However, there is a scarcity of studies on developing prediction models for persistent postoperative fever, particularly in the context of cardiac and great vessel surgeries.

This study presents a novel approach that incorporates a combination of nutritional and inflammatory markers, including NLR, MLR, SII, CONUT, and PNI, to predict the prognosis of cardiac surgery. These composite markers offer a more holistic assessment of patients’ inflammatory and nutritional status, surpassing the limitations of single laboratory markers. The utilisation of these composite markers proves advantageous in predicting clinical outcomes across a range of diseases [9]. The study revealed that CONUT score and MLR score were identified as independent risk factors for postoperative fever in patients undergoing surgery on large cardiac vessels. In the field of cardiovascular surgery, the CONUT score and the SII index serve as crucial reference markers for the treatment and management of patients, commonly utilised for prognostic predictions. A higher CONUT score is indicative of poor nutritional and immune status, which is associated with an unfavourable prognosis following cardiovascular surgery. Patients with a higher CONUT score exhibit a significantly lower overall survival rate. Therefore, maintaining a good preoperative nutritional status is crucial for improving the overall outcome of cardiovascular surgery [14]. Higher SII scores have been independently associated with an increased risk of major cardiovascular events (MACE), such as cardiac death and non-fatal stroke [15, 16], as well as postoperative delirium (POD) in elderly patients [17]. Furthermore, a 2022 study revealed a link between elevated MLR and a higher risk of POD. MLR could serve as a convenient and dependable marker for predicting POD in ICU patients undergoing cardiac surgery [18]. Moreover, MLR has demonstrated its utility in predicting patient outcomes in the context of breast cancer treatment and as a cost-effective predictor of acute kidney injury (AKI) following cardiac valve replacement surgery [19, 20]. These findings underscore the significance of preoperative comprehensive metrics as crucial biomarkers for evaluating risk and prognosis in patients undergoing specific cardiac surgeries. Nonetheless, this study did not establish a direct correlation between NLR, SII, PNI, and postoperative persistent fever.

The analysis presented in this article also emphasises several other key factors associated with persistent fever after cardiovascular surgery. These factors include prolonged operative time, blood transfusion during surgery, cardiopulmonary bypass (CPB), and patient-specific variables such as age and smoking history [21]. Among the identified risk factors for persistent postoperative fever following cardiovascular surgery, extended surgical duration, intraoperative blood transfusions, advanced age, and a history of smoking demonstrated significant associations, consistent with prior studies. Prolonged operative time likely indicates greater surgical complexity and prolonged tissue exposure, which may enhance inflammatory responses and susceptibility to infection. Extended cardiopulmonary bypass and ischemia-reperfusion injury are known to amplify systemic inflammatory cytokine release, thereby increasing the risk of fever and other postoperative complications [22]. Intraoperative transfusion of machine-washed red blood cells emerged as an independent predictor, suggesting that even filtered or processed blood products may not eliminate the pro-inflammatory effects associated with transfusion-related immunomodulation (TRIM). Several studies have shown that transfusions, particularly in cardiac surgery, correlate with increased inflammatory markers and postoperative morbidity [23, 24]. Older age has consistently been associated with altered immune responses and a heightened risk of postoperative complications. Age-related immunosenescence may contribute to persistent fever by impairing pathogen clearance while simultaneously promoting chronic low-grade inflammation [25]. A history of smoking was also identified as a significant predictor. Chronic smoking is known to induce systemic inflammation, impair mucociliary clearance, and elevate oxidative stress, thereby exacerbating postoperative inflammatory responses [26]. These physiological changes may predispose patients to prolonged febrile episodes even in the absence of overt infection. In addition, CPB has a significant impact on the incidence of postoperative fever. Numerous studies have reported that the rates of fever among cardiac surgery patients who underwent CPB range from 29.9 to 99% [3, 27, 28]. Postoperative fever following CPB is a multifaceted process with various contributing factors. It is commonly understood that blood interacts with the surfaces of tubes and oxygenators during CPB, triggering immune cell and coagulation system activation, which in turn leads to the release of cytokines and inflammatory mediators. Additionally, hypothermia is frequently utilised during CPB to safeguard organs, particularly the brain. However, rapid rewarming post-surgery can potentially cause an exaggerated response from the thermoregulatory centre, resulting in fever [29]. In an 18-year cohort study, researchers analysed the association between intraoperative nasopharyngeal temperature and mortality during coronary artery bypass grafting (CABG) surgery assisted by CPB [30]. The findings indicated that hypothermia could potentially benefit postoperative recovery, yet it could also result in adverse effects. This research underscores the significance of identifying optimal temperature management protocols during CPB. While the connection between intraoperative hypothermia and postoperative fever remains inconclusive, monitoring patients’ body temperature during cardiovascular surgery is crucial for predicting patient outcomes and complications. In investigating the risk factors for persistent fever following cardiovascular surgery, it is essential to consider the key aspects of management. Deviations in the execution of infection prevention and control protocols can contribute to prolonged postoperative fever. Numerous research studies have underscored the importance of precise surgical procedures and infection prevention strategies, including preoperative patient evaluation, antimicrobial prophylaxis, sterile techniques, and intraoperative infection control protocols, in reducing the likelihood of surgical site infections (SSIs) that could potentially prolong the duration of postoperative fever [31, 32]. Furthermore, we have enhanced communication and collaboration with cardiovascular surgeons. Initiatives such as the establishment of a comprehensive management system for the cleaning, disinfection, and sterilization of equipment and instruments, the standardization of sterile operating procedures for medical personnel, and the implementation of specialized training for both medical and support staff on the cleaning and disinfection of environmental surfaces have contributed to our successful outcomes. However, this study has several limitations. Firstly, it is a single-center study, which lacks evidence from a broader clinical setting. Secondly, in selecting variables, the study primarily focused on objective factors, such as preoperative and intraoperative conditions, thereby neglecting the potential impact of subjective factors, including various infection control measures and the influence of healthcare personnel. Thirdly, due to the influence of departmental performance evaluation indicators, such as the average length of hospital stay, the study only assessed the incidence of fever within seven days postoperatively. This lack of longer-term follow-up may introduce a degree of data bias.

## Conclusion

Several independent predictive factors for persistent fever following cardiovascular surgery include older age at admission, a history of smoking, a higher Controlling Nutritional Status Score (CONUT), an elevated Monocyte-to-Lymphocyte Ratio (MLR), a longer duration of surgery, the use of cardiopulmonary bypass, and intraoperative transfusion of machine-washed red blood cells. The predictive model developed in this study provides a more precise representation of clinical scenarios, enabling surgeons to estimate the likelihood of prolonged fever after these surgeries by evaluating patients’ medical histories and relevant examination results, thereby facilitating early intervention. This predictive capability underscores the need to adopt a targeted approach to patient management. It emphasises the importance of developing personalised risk mitigation strategies that are specifically tailored to the unique needs and circumstances of each patient.

## Data Availability

The data that support the findings of this study are available from the corresponding author upon reasonable request.
